# A critical review on oligometastatic disease: a radiation oncologist’s perspective

**DOI:** 10.1007/s12032-022-01788-8

**Published:** 2022-09-07

**Authors:** Pietro Pacifico, Riccardo Ray Colciago, Francesca De Felice, Luca Boldrini, Viola Salvestrini, Valerio Nardone, Isacco Desideri, Carlo Greco, Stefano Arcangeli

**Affiliations:** 1 School of Medicine and Surgery, University of Milan Bicocca, Milan, Italy; 2 Department of Radiation Oncology, Ospedale S. Gerardo, Via G. B. Pergolesi, 20900 Monza, MB Italy; 3 Department of Radiation Oncology, Fondazione IRCCS Istituto Nazionale Tumori, Milan, Italy; 4 Department of Radiotherapy, Policlinico Umberto I, Sapienza University of Rome, Rome, Italy; 5 Radiology, Radiation Oncology and Hematology Department, Fondazione Policlinico Universitario “Agostino Gemelli” IRCCS, Rome, Italy; 6 Radiation Oncology Unit, University of Florence, Florence, Italy; 7 Department of Precision Medicine, University of Campania “L. Vanvitelli”, Naples, Italy; 8 Department of Radiation Oncology, General Regional Hospital F. Miulli, Acquaviva delle Fonti, Bari, Italy; 9 Department of Radiation Oncology, Campus Bio-Medico University of Rome, Rome, Italy

**Keywords:** Oligometastasis, Oligometastatic disease, Prognostic factors, Stereotactic body radiation therapy

## Abstract

Since the first definition by Hellman and Weichselbaum in 1995, the concept of OligoMetastatic Disease (OMD) is a growing oncology field. It was hypothesized that OMD is a clinical temporal window between localized primary tumor and widespread metastases deserving of potentially curative treatment. In real-world clinical practice, OMD is a “spectrum of disease” that includes a highly heterogeneous population of patients with different prognosis. Metastasis directed therapy with local ablative treatment have proved to be a valid alternative to surgical approach. Stereotactic body radiation therapy demonstrated high local control rate and increased survival outcomes in this setting with a low rate of toxicity. However, there is a lack of consensus regarding many clinical, therapeutic, and prognostic aspects of this disease entity. In this review, we try to summarize the major critical features that could drive radiation oncologists toward a better selection of patients, treatments, and study endpoints. With the help of a set of practical questions, we aim to integrate the literature discussion.

## Introduction

To date, with the advent of new oncological strategies, such as immunotherapy and target therapy, cancer is increasingly becoming a chronic disease. Most deaths (up to 90%) from solid tumors are caused by metastasis [1, 2]. Moreover, cancer rates are estimated to increase by 47% in 2040 [3]. This means that Radiation Oncologists (RO) are expected to manage a growing number of metastatic patients. To meet this request, a deep understanding of the role of ablative treatments in this setting is of paramount importance.

Historically, surgical metastasectomy was the first local ablative approach that significantly enhanced clinical outcomes [4]. However, over the last decades advances in treatment planning, image guidance, target position reproducibility and on-line tracking, coupled with a compelling radiobiological rationale, have promoted the implementation of Stereotactic Body Radiation Therapy (SBRT), which has now become a valid treatment option especially for OligoMetastatic (OM) patients [5]. In a long term outcomes analysis of the randomized study SABR COMET, 21% of the patients with OligoMetastatic Disease (OMD) who underwent local Ablative RadioTherapy (ART) achieved a recurrence-free survival of more than 5 years [6]. As recommended elsewhere, as well as in a white paper by the Italian Association of Radiotherapy and Clinical Oncology, SBRT should therefore be offered to OM patients [7].

In this regard, RO have nowadays the opportunity to play a leading role among other oncological professionals. However, the vast heterogeneity in the clinical applications of Metastasis-Directed Therapy (MDT) has raised concerns about the use of SBRT, and efforts to homogenize it in daily clinical practice are needed.

The aim of this paper is to perform a critical review of the literature in order to critically assess the use of SBRT in the management of OM patients and to highlight challenges that are encountered in the implementation of this strategy. A set of practical questions was pre-formulated as a framework to generate discussion and promote a comprehensive exploration of subject matter with the aim of stimulating a critical thinking and handing out directions for future research and practice.

## Literature search

A literature review was performed between April 2021 and May 2022 using PubMed search engine with the terms oligometastasis and radiotherapy. Specific research questions were approached by searching for the following combinations of keywords: stereotactic body radiation therapy, SBRT, stereotactic ablative Radiation Therapy (RT), SABR, stereotactic radiosurgery, SRS, metastasis, prognosis, systemic treatment, immunotherapy, targeted therapy, hormonal therapy. Papers published in English were retained for their pertinence to the OMD multidisciplinary management. References lists were explored for relevant content and validity.

## Who is an “oligometastatic patient”?

Historically, the first definition of OMD was the statement by Hellman and Weichselbaum: a neoplasm that has spread to a single or a limited number of organs [8]. Dingemans et al. [9] tried to set specific threshold within this definition, suggesting a maximum of five metastases to a maximum of three organs.

However, this statement has several limitations. According to Ashworth et al. [10], 5-year Overall Survival (OS) of OM patients varies from 8.3% to 86%. Gutiontov et al. [11] argued that there are a lot of different factors which may account for variable outcomes: from clinical to genetic, from epigenetic to immunologic. Moreover, the European Society for Radiotherapy and Oncology (ESTRO) has recently published with the European Organization for Research and Treatment of Cancer (EORTC) a Delphi consensus recommendation for the characterization and classification of OMD [12]. In this consensus, a dynamic OM state model based on a decision tree of five binary disease characterization factors has been developed and proposed.

Despite the significant amount of data, a clear answer to the most relevant question on which OM patient benefits more from MDT is still pending.

## Who should be treated?

The lack of consensus regarding the definition of OMD does not favor the prognostic characterization of these patients. In the last 20 years, numerous studies have begun to detect factors that could be helpful in daily clinical practice for a better patients’ selection [13, 14]. We herein report the most important series.

### Number

Fode et al. [13] showed borderline significance (*p* = 0.049) for solitary metastasis in terms of OS, in a series of 321 OM patients treated with SBRT. In contrast, Franceschini et al. [14] reported no correlation between the number of metastasis (more than 1) and OS (*p* = 0.792) in a large cohort of 358 patients. Moreover, in various recent studies [14–17], the total number of metastases treated in OM patients does not seem to affect OS but only Progression-Free Survival (PFS). Phillips et al. [18] in a prospective II trial of SBRT in oligorecurrent prostate cancer showed that total consolidation of all Positron Emission Tomography (PET)-avid lesions resulted in a significant threefold increase in 6-month PFS and Distant Metastasis Free Survival (DMFS) with no grade 3 toxicity compared to those whose lesions were left untreated. These findings were mirrored in a phase II randomized trial of ART in patients with OM Castration Resistant Prostate Cancer (CRPC), where those who benefited most of the intensification of Abiraterone and SBRT in terms of complete biochemical response at 6 months were patients who underwent Prostate-Specific Membrane Antigen (PSMA) PET staging compared to non-PSMA PET staging [Odds Ratio 8.34 vs 1.32; *p* = 0.05)] [19]. Taken together these observations are consistent with the hypothesis that lesion consolidation by SBRT might alter the natural history of OMD by interfering with signals that promote further development of metastatic disease. Therefore, a numerical-based decision to withhold a local therapy may reduce the benefit of MDT in the treatment of OMD. Indeed, a recent ESTRO- American Society for Radiation Oncology consensus on OMD definition [20] claimed that “there is no biological evidence supporting the maximal number of metastases, or the maximal lesion size, that can be treated to provide clinical benefit”.

### Site

Location of metastases is a controversial prognostic factor. Franceschini et al. [14] reported a strong correlation between the presence of lung or nodal metastases and longer OS (*p* = 0.001), whereas patients with liver or brain localizations were found independent predictors of any progression and poorer OS. In a large cohort of 270 patients with OM-ColoRectal Cancer (OM-CRC), Franzese et al. [17] showed a longer OS of lung metastasis compared with non-lung sites. The incidence of brain and liver metastases has increased with the advent of Magnetic Resonance Imaging, as advanced imaging has definitely improved our ability to detect small lesions earlier in the course of disease. This observation, along with more shared and homogeneous treatment regimens in the RO community, could allow us to achieve better results also in this setting. The ESTRO-EORTC consensus statement [12] about the definition of OMD did not distinguish extracranial from intracranial metastases, and recommended that patients with intracranial metastases should not be excluded from trials on OM patients. A recent review published by Suh JH et al. [21] highlighted that a subset of brain metastasis patients may live for years after diagnosis, especially those with “limited” intracranial disease (up to 4 metastasis), from specific primary histologies (e.g., breast cancer) and with targetable molecular alterations [22]. These findings emphasize the prognostic relevance of metastases location, and namely that lung lesions are associated with a better prognosis. However, the site of the metastasis as prognostic factor must be analysed in close relation with the primary histology, the number of lesions, and their size.

### Size

The prognostic relevance of metastases’ size is still unclear. In a recent multicenter retrospective study with 1378 patients published by Yamamoto et al. [23], a maximum OM tumor diameter (per 1-cm increase) has showed a strong correlation (*p* < 0.001) with OS. However, Girard et al. [24] compared tumor size and detectability by number of tumors Doubling Times (DT). The authors hypothesized that the probability that even undetectable lesions are present increases as small as the metastases are found. More specifically, they suggest that only a single metastasis that has reached a diameter of 32 mm (34 of DT) would have an 80% probability of being truly solitary. Moreover, three large cohort studies [13, 17, 25] reported that a metastasis cut-off of 30 mm significantly correlated with better OS. Franzese et al. [17] in patients with OM-CRC showed that a Clinical Target Volume > 30 mm was associated with worse prognosis (*p* = 0.03). In a recently published multicenter large retrospective database on the personalization of Stereotactic ABlative Radiotherapy (SABR) use in lung metastases from CRC (LaIT-SABR study) [26], a correlation between tumor size and the development of the polymetastatic disease was demonstrated, other than metastases number. Specifically, patients with metastases diameter exceeding 20 mm and with > 3 metastases had a significantly short time to polymetastatic conversion. These findings support the presence of a survival prognostic size cute-off.

### Tumor markers

Although no biomarkers that can differentiate between the oligometastatic and the polymetastatic state have been so far validated, some are routinely used as prognostic factors especially in CRC and prostate cancer. A short Prostate-Specific Antigen (PSA) DT is known to predict both the development of metastasis and prostate cancer-specific mortality in patients who underwent primary treatment [27]. PSA kinetics may be an important predictor of mortality in recurrent prostate cancer [28] and PSA DT is also a strong predictor of metastasis and survival in non-metastatic CRPC [29].

However, in the OM setting, PSA DT was not predictive of OS and PFS but only of Androgen Deprivation Therapy Free Survival (ADTFS) [17, 30, 31]. In metastatic CRC Thompson et al. [32], reported that pre-SBRT Carcinoembryonic Antigen (CEA) was a significant predictor of better OS with a predictive cut-off of 100 ng/ml.

In recent years, novel biomarkers are emerging through the use of liquid biopsy. The blood test of Circulating Tumor Cells (CTCs) and Circulating Tumor DNA (ctDNA) showed a prognostic value in OMD. In a prospective analysis of 43 patients with various primitive histologies, a lack of CTC clearance to ≤ 15/ml after 100 days by the end of SBRT was associated with progression of the irradiated lesion [33]. Lebow et al. [34] analyzed 820 patients with advanced Non-Small Cell Lung cancer (NSCLC) who underwent liquid biopsy with plasma next-generation sequencing of ctDNA. OMD was associated with a lower rate of ctDNA detection compared to polymetastatic disease, identifying a strong correlation between number of disease site and ctDNA. In the near future, these novel biomarkers will likely be integrated into a more comprehensive algorithm for defining OMD other than the number of metastases, and will represent a helpful tool to optimize the treatment strategy.

### Timing

Disease-Free Interval (DFI), defined as the time between primary diagnosis and the detection of the first metastasis, is a deep-analyzed prognostic factor. Alongi et al. [35] reported a longer OS for DFI < 30 months for lung oligometastases. Similar data were found in a recursive partitioning-based analysis by Franzese et al. [17], who observed a DFI cut-off of 34 months in OM-Prostate Cancer. These data suggest that DFI is a promising parameter to detect which OM patients would benefit most from SBRT.

In a multi-institutional database recursive partitioning analysis reported by Chen et al. [36], patients with extracranial OM disease and metachronous presentation over 24 months showed a better OS (36.5 vs 17.1 months) compared with metachronous presentation ≤ 24 months. Interestingly, the time factor influenced OS only in case of extrapulmonary disease and specific histologies (NSCLC, Head and Neck, Breast triple negative, Melanoma, Sarcoma).

The EORTC-ESTRO OligoCare consensus recommendation differentiated into synchronous versus metachronous states, according to the interval between primary cancer diagnosis and development of OMD. As such, OMD is defined as synchronous if metastases are detected within 6 months from the initial diagnosis, and metachronous in case of a later appearance (at least 6 months) from the initial diagnosis [12]. There’s no shared consensus on defining the time point for synchronous/metachronous and the prognostic implications still remain unclear. Moreover, there are a number of studies with controversial data about the real prognostic impact on OS of metastatic disease timing. Three large retrospective studies reported no correlation between OM presentation (synchronous/metachronous) and survival outcomes (OS and PFS) [14, 15, 37]. A retrospective analysis of 194 patients with synchronous OM-NSCLC treated with MDT (radiotherapy, surgical therapy or other local ablative therapy) [38] showed a 5-years OS rate of 27–32% with a median follow-up of 52 months. In contrast, Fode et al. [13] show a favorable prognosis for metachronous metastases (*p* = 0.02) in a population represented for 98% of a location confined to a single organ (lung or liver). Obviously, the time factor is closely related to the ability of the imaging modalities to detect even the smallest lesions for a correct definition of a synchronous vs metachronous disease. Modern and more accurate staging systems (e.g., PET PSMA or liquid biopsy) are needed to consolidate DFI and OM presentation as prognostic factors. A recent prospective phase II trial testing the OM hypothesis in patients with positive PET PSMA for prostate cancer recurrence treated with MDT found a biochemical complete response rate of 22% without the use of Androgen Deprivation Therapy (ADT) or other therapies [39].

### Prior systemic therapy

The numbers of systemic therapy lines prior to MDT could heavily influence the local response and survival outcomes in OM-CRC patients. Three large cohort studies found that numbers of systemic lines administered prior to SBRT have a negative influence on OS and PFS, suggesting that tumor cells surviving after ChemoTherapy (CT) acquire an improved DNA repair capacity, switching to a more radioresistant phenotype [17, 32, 40]. In particular, Klement et al. [40] showed a consistent dose–response relationship among pre-SBRT chemotherapy and local control. Unlike chemotherapy-naïve metastases, SBRT treatment with prior systemic therapy required a Biologically Effective Dose (BED) of more than 209 Gy_10_ to achieve 90% local control at 2 years. Furthermore, patients who received more than 3 lines of chemotherapy had the worst outcomes in terms of PFS rates (26% vs 55%). Conversely, immunotherapy seems to enhance the effect of radiotherapy, in particular at high doses per fraction. A recent retrospective analysis by Kroeze et al. [41] of 108 patients with oligoprogressive or polyprogressive disease treated with multiple line of Target Therapy (TT) or ImmunoTherapy (IT) and MDT shows a significant correlation between previous lines of systemic therapy and PFS (*p* = 0.033) at multivariate analysis. This data suggest that the reduced efficacy of subsequent lines of systemic therapy (CT or TT/IT) could drive distant progression. In this scenario, MDT could delay the systemic line therapy-switch and therefore play an increasingly important role.

### Histology

The primary histology seems to be less correlated with local control, maybe due to the current wide use of ablative doses. Franceschini et al. [14] reported no significant association between any survival outcomes and tumor histology with a median BED administered of 105 Gy. However, some studies suggest a sort of radioresistance for CRC metastases [35, 40]. The presence of radioresistant histologies is largely due to their biological characteristics (e.g., high proportion of hypoxic cells) but also to a clonal selection caused by several lines of pre-RT chemotherapies [40]. Nevertheless, a strong evidence of correlation with OS exists for breast or prostate primary tumors, both showing better OS compared to the other tumors [42, 43]. Milano et al. [42] showed a significant discrepancy in terms of OS (*p* < 0.00001) for OM-breast cancer treated with a BED of 100 Gy_10_, with a 6-year OS rate of 47% compared to 9% for non-breast-histology. Franzese et al. [43] reported a 3-years OS rate of 88% in a population of OM-prostate cancer treated with a median BED of 157.5 Gy. In a recent update of a multi-institutional database consisting of 1033 patients with OM (≤ 5 metastases) treated with SBRT between 2006 and 2017 [44], conditional PFS stratified by primary site significantly increased over time for patients with CRC, breast and kidney cancer, remained stable for NSCLC and kidney cancer and significantly decreased for prostate, breast and CRC. Although primary histology remains a strong prognostic factor in terms of OS, regardless of the dose used, a routine use of ART doses is required to ensure a high rate of local control of the disease.

### Performance status

The prognostic value of Performance Status (PS) is often underestimated. Most studies have demonstrated a strong correlation between OS and PS [13, 23]. In contrast, others large cohort studies [14, 17, 32] did not showed a significant statistical correlation at multivariate analysis, but only at univariate analysis. PS score incorporates a series of independents biological parameters of the patient (e.g., age, cognitive impairment, sarcopenia, malnutrition, advanced disease, comorbidities, pain cancer etc.) that heavily influence OS. Each of these parameters must be assessed individually based on the location of the disease and the primary histology of the tumor (e.g., cognitive impairment for brain metastasis, age and comorbidities for prostate cancer, pain for bone locations, sarcopenia and malnutrition for head and neck tumors, etc.). PS score should always be evaluated before offering treatments with a high risk of toxicity or less established therapeutic approaches. More personalized prognostic score based on activities of daily life and metastases site of tumors are required.

## Which dose to prescribe?

The choice of prescription dose still remains controversial, due to the vast treatment heterogeneity described in literature and the lack of robust randomized controlled trials (RCT).

The first dose-guiding step is to define a common term to compare the different possible fractionation schedules. BED, defined as BED = D × [1 + d/(α/β)] (D is total dose delivered; d is dose per fraction; α/β = 10 for malignant lesion), is commonly used for isoeffective dose calculation.

Starting from retrospective data, Kobiela et al. [45] reported that BED used in literature varied from 40.5 to 265 Gy, and concluded that it is challenging to find an ideal dose. Nevertheless, they observed that a higher BED correlates with higher local control in the oligometastatic CRC setting. Similarly, Chang et al. [46] found that BED ≥ 75 Gy for OM-CRC patients is related to better local control at 18 months compared to BED < 75 Gy (80% VS 31%, *p* = 0.00001). Jing Yu et al. [47] showed that in OM-CRC a BED ≥ 100 Gy was associated to a significantly better 1-year local control than BED < 100 Gy (94.4% VS 63.2%; *p* = 0.022) and 1-year OS (100% VS 73.4%; *p* = 0.028). The same cut-off was proposed by Guckenberger et al. [48], who observed better local control rates at 36 months for lung tumors when a BED > 100 Gy was reached, compared to BED < 100 Gy (89 VS 62%, *p* = 0.00001). Nicosia et al. [26] published the largest retrospective series of lung OM-CRC treated with SBRT. BED ≥ 125 Gy drastically reduced the risk of local progression both at univariate and multivariate analyses compared to BED < 125 Gy (multivariate HR 0.24, 95%CI 0.11–0.51; *p* = 0.000). Moreover, higher BED was associated with a significantly longer time to polymetastatic conversion as compared to lower BED. Lastly, Burkon et al. [49] found that in OMD BED_10_ values of 150–170 Gy compared to 100–150 Gy were independent positive prognostic factors for local PFS (Hazard Ratio 0.25), confirming that ablative doses are effective regardless the OMD primary histology and location.

However, the most important RCTs summarized in Table 1, seem to suggest a different perspective. In the whole court of OM-NSCLC patients analyzed by Gomez et al. [50] a significative improvement of PFS and OS was demonstrated when RT was added to maintenance therapy, even if only few courses actually delivered a BED > 100 Gy.

**Table 1:** Randomized controlled trials of MDT

Study	Phase	Type of cancer	Intervention	BED (Gy)	Outcomes
Gomez et al. [50]	II	NSCLC with 1–3 metastases	Maintenance therapy with or without local consolidative therapy	39–119	PFS 14.2 vs 4.4 months (*p* = 0.022). OS 41.2 vs 17.0 months (*p* = 0.017)
Iynegar et al. [51]	II	NSCLC with 1–5 metastases	Maintenance chemotherapy with or without SBRT to all sites	44–80	PFS 9.7 vs 3.5 months (*p* = 0.01)
Palma et al. [52]	II	All histologies with 1–5 metastases	SBRT to all metastatic sites vs palliative standard of care	50–151	OS 53 vs 28 months (*p* = 0.008)
Harrow et al. [53]					PFS 12 vs 5.4 months (*p* < 0.001)
Ost et al. [54]	II	Recurrent prostate cancer with 1–3 metastases	Maintenance chemotherapy with or without SBRT to all sites	60	ADT-free survival 21 vs 13 months (*p* = 0.11)

*BED* Biologically Effective Dose, *NSCLC* Non-Small Cell Lung Cancer, *PFS* Progression-Free Survival, *OS* Overall Survival, *ADT* Androgen Deprivation Therapy

Iynegar et al. [51] treated with maintenance chemotherapy ± SBRT to all sites of disease 29 OM-NSCLC patients. Although the maximum BED used was 80 Gy, local ablative treatments led to a longer PFS of 9.7 compared to 3.5 months (*p* = 0.01) of the standard therapy.

Palma et al. [52] compared SBRT (BED ranging from 50 to 157 Gy) with palliative standard of care to all metastatic sites in patients with different primary tumors. Results showed an improvement of OS to 41 vs 28 months (*p* = 0.09) and of PFS to 12 vs 6 months (*p* = 0.0012), and a recent update [53] confirmed durable improvements in OS and PFS, and no major toxicity with extended follow-up. An interim analysis of first 1004 patients enrolled in the OligoCare trial, aimed at identifying patient, tumor, staging and treatment characteristics that affect OS after radical radiotherapy for OM breast, CRC, prostate, and NSCLC disease, showed a large heterogeneity in terms of median dose per fraction: 9.7 Gy (range 3–39); median number of fractions: 5 (range 1–12); and median BED: 74.4 Gy (range 40.4–297.3). Notwithstanding, primary tumor, location of oligometastases and lesions’ size were significantly associated with SBRT dose [*presented at ISRS 2022*].

In conclusion, while retrospective data suggest improved local control of the targeted lesions with a minimum of 100 Gy BED_10_, provided that normal tissues’ dose-volume constraints are fulfilled, there are not sufficient evidence to address dose and BED in this setting, and RCTs show that even treatments with BED < 100 Gy are associated with excellent oncological outcomes. Furthermore, it must be acknowledged that in studies where OMD has emerged as a limited resistance in the context of systemic therapy, generally lower radiation doses have been used compared to those focused on synchronous or metachronous OMD. A possible interpretation of these observations postulates that the driver of prognosis is likely the distant rather than the local control, which can be achieved with the ablation of every metastatic site regardless the use of a very high BED. On the other hand, accumulating data make the delivery of a BED 100 a reasonable goal, if safely treatable, until further evidence emerges.

## What is the aim?

There is not a univocal main goal of MDT. As recently described by Gutiontov et al. [10], OM represents a “spectrum of disease” containing different tumors, at different stages, with different biologic hallmarks and therefore with different prognosis. Thus, due to this selection bias, is it still uncertain to establish if MTD impacts clinical outcomes, as well as to define which endpoints can be considered valid, whether survival or time to polymetastatic progression or time to NExt Systemic Therapy (NEST).

Ideally, study objectives should be adapted to both the primary histology and to the OM subtype classification. As an example, ADTFS seems to be an optimal endpoint for prostate cancer, as hormone therapy negatively affects patients' quality of life and increases the risk of cardiovascular events.

On the other hand, OS remains a robust endpoint in oligorecurrent or de-novo OM lung cancer or CRC. Instead, in the setting of oligoprogressive disease NEST seems to be a better surrogate endpoint for QoL and PFS.

**Fig. 1:**
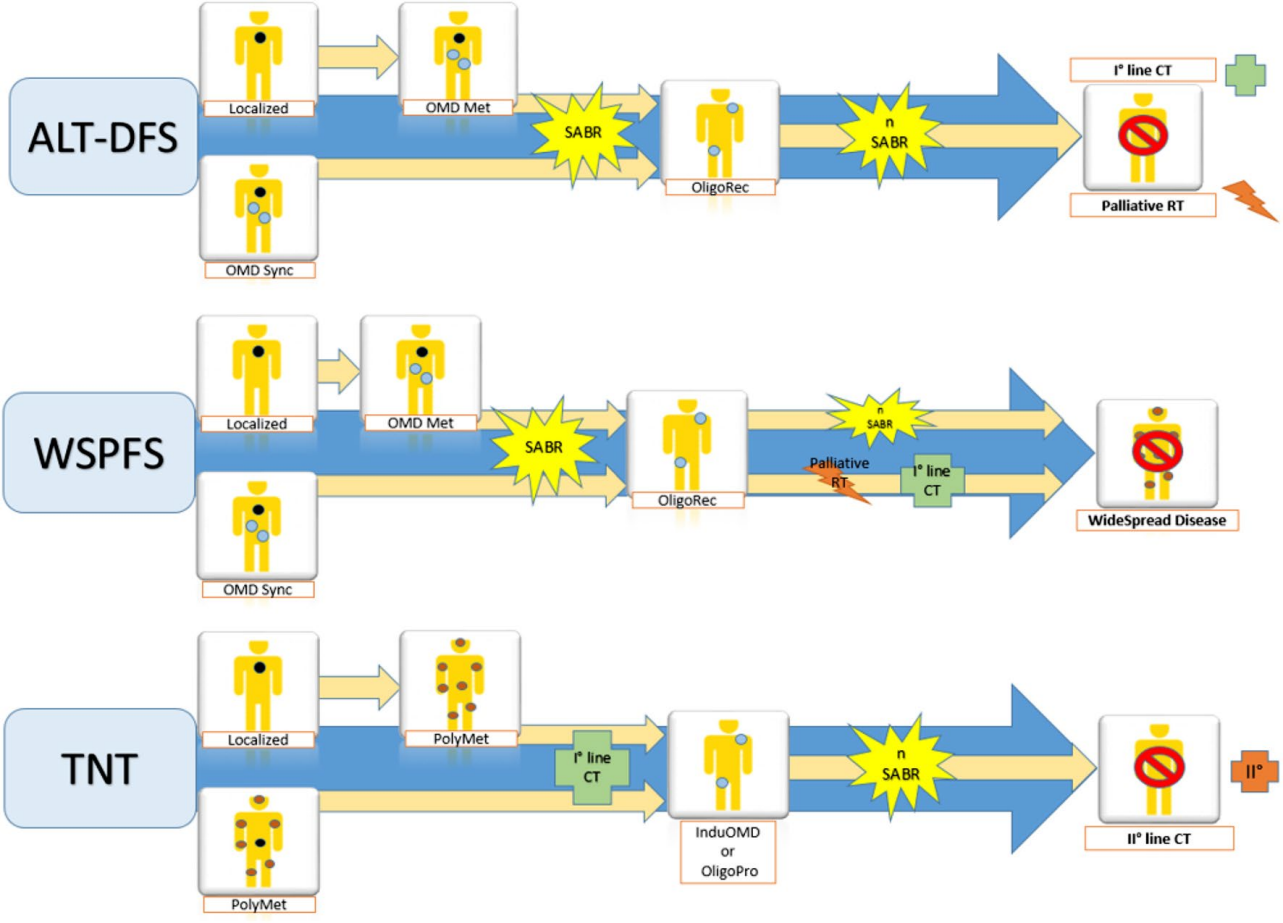
Novel endpoints for OMD. *ALT* Ablative Local Treatment-adjusted Disease-Free Survival, *WSPFS* widespread Progression-Free Survival, *TNT or NEST* Time to New Systemic treatment

Finally, Loi M. et al. [55] suggested Ablative Local Treatment (ALT)-adjusted PFS (time from first ALT to systemic treatment or best supportive care), widespread PFS (time from oligometastatic presentation to metastatic dissemination) and Systemic Therapy plus ALT-adjusted PFS (time from chemotherapy initiation to further chemotherapy line) as novel endpoints (Fig. 1).

## Summing up

OMD is increasingly described as a clinical temporal window of each metastatic tumor. This review has focused on the identification of the main OM disease features and clinical applications of MDT.

Although the number of metastatic lesions needed to define the OM presentation should not be interpreted as the sole parameter to delve into patients’ disease, it still remains the key factor that drives the decision-making process, and namely with a maximum of 5 lesions.

Despite its weakness, it continues to be routinely used among the inclusion criteria in RCT, mainly due to the absence of other validated selection parameters. Based on the previously discussed literature data, Table 2 summarizes the main OM disease characteristics which are supposed to be associated with better clinical outcomes.

**Table 2:** Resume of cut-off values of prognostic factor for OMD in retrospective studies

Prognostic factors	Cut-off values	References	Outcomes
Size	Pulmonary metastasis: 30 mm	Fode et al. [13]	OS, LPFS
	OM-CRC: 20-30 mm	Franzese et al. [17]	
		Sharma et al. [25]	
		Nicosia et al. [26]	
Number	1–5	Fode et al. [13]	OS, tPMC
	OM-CRC: 3	Franceschini et al. [14]	
		Klement et al. [15]	
		Ricardi et al. [16]	
		Franzese et al. [17]	
		Nicosia et al. [26]	
Site	Lung metastasis	Franceschini et al. [14]	OS
	OM-PC: Bone only	Franzese et al. [17]	
		Chen et al. [44]	
DFI	Pulmonary metastasis: 30 months	Franzese et al. [17]	OS, PFS
	OM-PC: 24–34 months	Alongi et al. [35]	
	OM-CRC: 30 months	Chen et al. [36]	
	EP-OM other histologies^a^: 24 months	Chen et al. [44]	
Markers	OM-CRC: CEA < 100 ng/ml	Thompson et al. [32]	OS, PFS
	NSCLC: CTC clearance to ≤ 15/ml	Lebow et al. [34]	
Prior systemic therapy	OM-CRC: < 2 line	Franzese et al. [17]	OS
		Thompson et al. [32]	
		Klement et al. [40]	
Primary site	Breast, prostate	Milano et al. [42]	OS
		Chen et al. [44]	
PS	0–1	Fode et al. [13]	OS
		Yamamoto et al. [23]	

*OMD* Oligometastatic-Disease, *LPFS* Local Progresison Free Survival, *tPMC* time to PolyMetastatic Conversion, *DFI* Disease-Free Interval, *PS* Performance Status, *OM* Oligometastatic, *CRC* Colorectal Cancer, *PC* Prostate Cancer, *EP* Extrapulmonary, *CEA* Carcinoembryonic Antigen, *OS* Overall Survival, *PFS* Progression-Free Survival

^a^NSCLC, H&N, Breast triple negative, Melanoma, Sarcoma

For instance, the definition of site, size, DFI, prior-SBRT chemotherapy, and CEA play a leading role as prognostic factors, supporting the selection of OM-CRC patients. Noteworthy, the number of metastases and timing of OM presentation (synchronous or metachronous) have not yet reached a broad consensus as independent prognostic values for most histologies.

As a general practical guide to SBRT prescription, based on the available evidence treatments can be delivered reaching a BED_10_ of at least 100 Gy, provided that normal tissues tolerance is not exceeded. However lower BED should not preclude the opportunity to attempt a MDT approach to all sites of OMD, especially in combination with concurrent systemic therapy or immunotherapy, or in the case of prostate tumors whose high sensitivity to dose per fraction is supposed to increase the therapeutic gain. New shared clinical endpoints are certainly needed to achieve a better homogeneity in the results of the next RCTs, with specific focus on the histology (indolent versus aggressive histologies) and the subtype of clinical presentation (De novo OM disease versus Oligoprogressive disease). Further efforts should be spent in investigating the role of translational biomarkers in order to better define OM disease (CTCc or ctDNA), as well as the effect of SBRT in the immune system.

Surgical MDT remains a primary therapeutic option for selected patients with resectable OMD, typically with lung or hepatic location, synchronous presentation, multiple metastases in the same lobe and good performance status [56, 57]. In a recent retrospective analysis of the M.D. Anderson Cancer Center [58], the authors examined outcomes after surgical MDT with RT (BED 55–60 Gy_10_) used as a benchmark comparator, and showed excellent outcomes in synchronous oligometastatic NSCLC with a median OS of 55.2 months vs 23.4 months, respectively. However, surgical patients were younger and had lower intrathoracic disease burden, which might have favoured surgery over RT. No randomized data exist about a direct comparison between SBRT and surgical MDT in OMD, and in retrospective studies SBRT was often offered in patients unfit or unwilling to undergo a surgical treatment resulting in selection bias.

Table 3 provides a summary of the ongoing phase III RCTs which are expected to have a relevant impact on our clinical practice in the field of OMD.

**Table 3:** OMD on-going phase III randomized controlled trials

Study	Phase	Type of cancer	Intervention	Estimated completion date	Primary endpoint
NCT05278052	III	NSCLC	Standard maintenance therapy + SBRT	2028	2 year—OS
			VS		
			Standard maintenance therapy alone		
NCT05377047	III	Breast cancer	SBRT to all sites	2027	3 year—OS
			VS		
			Standard first line systemic therapy		
NCT04983095	III	Prostate cancer	SBRT to all sites + standard treatment	2029	Failure-free survival
			VS		
			Standard treatment		
NCT04498767	III	Solid tumors	SBRT to all sites	2030	OS
			VS		
			Palliative RT		
NCT04495309	III	Breast cancer	SBRT to all sites + Standard treatment	2025	PFS and QoL
			VS		
			Standard treatment		
NCT02417662	III	NSCLC	SBRT to all sites + Standard treatment	2022	3 year—OS
			VS		
			Standard treatment alone		
NCT04599686	III	Prostate cancer	SBRT to all sites	2025	1 year—ADT-free survival
			VS		
			ADT		
NCT04115007	III	Prostate Cancer	SBRT to all sites + Standard treatment	2027	Castration-resistant prostate cancer free survival
			VS		
			Standard treatment		
NCT04646564	III	Breast cancer	SBRT to all sites + Standard treatment	2026	2 year—PFS
			VS		
			Standard treatment		
NCT03862911	III	Solid tumors	SBRT to all sites + Standard treatment	2028	5 year—OS
			VS		
			Standard treatment		
NCT03784755	III	Prostate cancer	SBRT to all metastatic lesions and primary tumor + Standard treatment	2025	Failure-free survival
			VS		
			SBRT to primary tumor + Standard treatment		
NCT03721341	III	Solid tumors	SBRT to all sites + Standard treatment	2029	OS
			VS		
			Standard treatment		
NCT05209243	III	Prostate cancer	SBRT to all metastatic sites + ADT + Standard treatment + RT to primary tumor	2026	2 year—PFS
			VS		
			ADT + RT to primary tumor + Second generation hormonal treatment		
NCT03827577	III	NSCLC	SBRT to all sites + Lung resection + Standard treatment	2022	5 year—OS
			VS		
			Standard treatment		
NCT05352178	III	Prostate cancer	SBRT to all sites	2032	5 year—Poly metastatic free survival
			VS		
			SBRT to all sites + ADT		

*NSCLC* Non-Small Cell Lung Cancer, *PFS* Progression Free Survival, *OS* Overall Survival, *ADT* Androgen Deprivation Therapy, *RT* Radiation Therapy, *QoL* Quality of Life

## Conclusions

Cancer treatment decision-making for OM patients is complex and radiotherapy plays a significant role in this setting.

SBRT seems to be associated with improved clinical outcomes if delivered with a BED > 100 Gy, up to five lesions with a maximum diameter of 30 mm, with a DFI of at least 24 months and a PS of 0–1. Further well-designed RCTs are needed to confirm these findings and provide evidence-based support for the best OM patients care.

## References

[1] Dillekås H, Rogers MS, Straume O (2019). Are 90% of deaths from cancer caused by metastases?. Cancer Med.

[2] Seyfried TN, Huysentruyt LC (2013). On the origin of cancer metastasis. Crit Rev Oncog.

[3] Sung H, Ferlay J, Siegel RL (2021). Global Cancer Statistics 2020: GLOBOCAN estimates of incidence and mortality worldwide for 36 cancers in 185 Countries. CA Cancer J Clin.

[4] Pastorino U, Buyse M, Friedel G (1997). International prospet of lung metastases. Long-term results of lung metastasectomy: prognostic analyses based on 5206 cases. J Thorac Cardiovasc Surg.

[5] Alongi F, Arcangeli S, Filippi AR (2012). Review and uses of stereotactic body radiation therapy for oligometastases. Oncologist.

[6] Harrow S, Palma DA, Olson R (2022). Stereotactic Radiation for the Comprehensive Treatment of Oligometastases (SABR-COMET)—extended long-term outcomes. Int J Radiat Oncol Biol Phys.

[7] Alongi F, Nicosia L, Arcangeli S, et al. White Paper AIRO a cura del gruppo di studio “biologia e trattamento delle oligometastasi” e del “gruppo di studio uro-oncologico”—2021 Feb 1

[8] Hellman S, Weichselbaum RR (1995). Oligometastases. J Clin Oncol.

[9] Dingemans AC, Hendriks LEL, Berghmans T (2019). Definition of synchronous oligometastatic non-small cell lung cancer—a consensus report. J Thorac Oncol.

[10] Ashworth A, Rodrigues G, Boldt G (2013). Is there an oligometastatic state in non-small cell lung cancer? A systematic review of the literature. Lung Cancer.

[11] Gutiontov SI, Pitroda SP, Tran PT (2021). (Oligo)metastasis as a spectrum of disease. Cancer Res.

[12] Guckenberger M, Lievens Y, Bouma AB (2020). Characterisation and classification of oligometastatic disease: a European Society for radiotherapy and oncology and European Organisation for research and treatment of cancer consensus recommendation. Lancet Oncol.

[13] Fode MM, Høyer M (2015). Survival and prognostic factors in 321 patients treated with stereotactic body radiotherapy for oligo-metastases. Radiother Oncol.

[14] Franceschini D, De Rose F, Franzese C (2019). Predictive factors for response and survival in a cohort of oligometastatic patients treated with stereotactic body radiation therapy. Int J Radiat Oncol Biol Phys.

[15] Klement RJ, Hoerner-Rieber J, Adebahr S (2018). Stereotactic body radiotherapy (SBRT) for multiple pulmonary oligometastases: analysis of number and timing of repeat SBRT as impact factors on treatment safety and efficacy. Radiother Oncol.

[16] Ricardi U, Filippi AR, Guarneri A (2012). Stereotactic body radiation therapy for lung metastases. Lung Cancer.

[17] Franzese C, Comito T, Toska E (2019). Predictive factors for survival of oligometastatic colorectal cancer treated with stereotactic body radiation therapy. Radiother Oncol.

[18] Phillips R, Shi WY, Deek M (2020). Outcomes of observation vs stereotactic ablative radiation for oligometastatic prostate cancer: the ORIOLE phase 2 randomized clinical trial. JAMA Oncol.

[19] Francolini G, Detti B, Di Cataldo V, et al. “ARTO trial-(NCT03449719)”; Abstract ESTRO 2022

[20] Lievens Y, Guckenberger M, Gomez D (2020). Defining oligometastatic disease from a radiation oncology perspective: an ESTRO-ASTRO consensus document. Radiother Oncol.

[21] Suh JH, Kotecha R, Chao ST (2020). Current approaches to the management of brain metastases. Nat Rev Clin Oncol.

[22] Chaung KV, Sloan AE, Choi S (2021). Limited brain metastases: a narrative review. Ann Palliat Med.

[23] Yamamoto T, Niibe Y, Aoki M (2020). Analyses of the local control of pulmonary oligometastases after stereotactic body radiotherapy and the impact of local control on survival. BMC Cancer.

[24] Girard P, Gossot D, Mariolo A (2021). Oligometastases for clinicians: size matters. J Clin Oncol.

[25] Sharma A, Duijm M, Oomen-de Hoop E (2019). Survival and prognostic factors of pulmonary oligometastases treated with stereotactic body radiotherapy. Acta Oncol.

[26] Nicosia L, Franceschini D, Perrone-Congedi F (2021). A multicenter LArge retrospectIve daTabase on the personalization of stereotactic ABlative radiotherapy use in lung metastases from colon-rectal cancer: The LaIT-SABR study. Radiother Oncol.

[27] Jackson WC, Johnson SB, Li D (2013). A prostate-specific antigen doubling time of <6 months is prognostic for metastasis and prostate cancer-specific death for patients receiving salvage radiation therapy post radical prostatectomy. Radiat Oncol.

[28] Vickers AJ, Brewster SF (2012). PSA velocity and doubling time in diagnosis and prognosis of prostate cancer. Br J Med Surg Urol.

[29] Howard LE, Moreira DM, De Hoedt A (2017). Thresholds for PSA doubling time in men with non-metastatic castration-resistant prostate cancer. BJU Int.

[30] Decaestecker K, De Meerleer G, Lambert B (2014). Repeated stereotactic body radiotherapy for oligometastatic prostate cancer recurrence. Radiat Oncol.

[31] Kneebone A, Hruby G, Ainsworth H (2018). Stereotactic body radiotherapy for oligometastatic prostate cancer detected via prostate-specific membrane antigen positron emission tomography. Eur Urol Oncol.

[32] Thompson R, Cheung P, Chu W (2020). Outcomes of extra-cranial stereotactic body radiotherapy for metastatic colorectal cancer: dose and site of metastases matter. Radiother Oncol.

[33] Sud S, Hall J, Tan X (2021). Prospective characterization of circulating tumor cell kinetics in patients with oligometastatic disease receiving definitive radiation therapy. Int J Radiat Oncol Biol Phys.

[34] Lebow ES, Murciano-Goroff Y, Razavi P (2020). Circulating tumor DNA as a biomarker in oligometastatic non-small cell lung cancer. Int J Radiat Oncol Biol Phys.

[35] Alongi F, Mazzola R, Figlia V (2018). Stereotactic body radiotherapy for lung oligometastases: Literature review according to PICO criteria. Tumori.

[36] Chen H, Poon I, Atenafu EG (2021). Development of a prognostic model for overall survival in patients with extracranial oligometastatic disease treated with stereotactic body radiation therapy. Int J Radiat Oncol Biol Phys.

[37] Sharma A, Baker S, Duijm M (2020). Prognostic factors for local control and survival for inoperable pulmonary colorectal oligometastases treated with stereotactic body radiotherapy. Radiother Oncol.

[38] Mitchell KG, Farooqi A, Ludmir EB (2020). Improved overall survival with comprehensive local consolidative therapy in synchronous oligometastatic non-small-cell lung cancer. Clin Lung Cancer.

[39] Glicksman RM, Metser U, Vines D (2021). Curative-intent metastasis-directed therapies for molecularly-defined oligorecurrent prostate cancer: a prospective phase II trial testing the oligometastasis hypothesis. Eur Urol.

[40] Klement RJ, Guckenberger M, Alheid H (2017). Stereotactic body radiotherapy for oligo-metastatic liver disease—influence of pre-treatment chemotherapy and histology on local tumor control. Radiother Oncol.

[41] Kroeze SGC, Schaule J, Fritz C (2021). Metastasis directed stereotactic radiotherapy in NSCLC patients progressing under targeted- or immunotherapy: efficacy and safety reporting from the ‘TOaSTT’ database. Radiat Oncol.

[42] Milano MT, Katz AW, Zhang H (2012). Oligometastases treated with stereotactic body radiotherapy: long-term follow-up of prospective study. Int J Radiat Oncol Biol Phys.

[43] Franzese C, Di Brina L, D'Agostino G (2019). Predictive factors for survival outcomes of oligometastatic prostate cancer patients treated with metastases-directed therapy: a recursive partitioning-based analysis. J Cancer Res Clin Oncol.

[44] Chen H, Badellino S, Biswas T (2022). Conditional survival of extracranial oligometastatic patients treated with stereotactic Body Radiation Therapy (SBRT): an International Consortium Study. Int J Radiat Oncol Biol Phys.

[45] Kobiela J, Spychalski P, Marvaso G (2018). Ablative stereotactic radiotherapy for oligometastatic colorectal cancer: systematic review. Crit Rev Oncol Hematol.

[46] Chang DT, Swaminath A, Kozak M (2011). Stereotactic body radiotherapy for colorectal liver metastases: a pooled analysis. Cancer.

[47] Yu J, Li N, Tang Y (2019). Outcomes after hypofractionated stereotactic radiotherapy for colorectal cancer oligometastases. J Surg Oncol.

[48] Guckenberger M, Wulf J, Mueller G (2009). Dose-response relationship for image-guided stereotactic body radiotherapy of pulmonary tumors: relevance of 4D dose calculation. Int J Radiat Oncol Biol Phys.

[49] Burkon P, Kazda T, Pospisil P (2019). Ablative dose stereotactic body radiation therapy for oligometastatic disease: a prospective single institution study. Neoplasma.

[50] Gomez DR, Tang C, Zhang J (2019). Local consolidative therapy vs. maintenance therapy or observation for patients with oligometastatic non-small-cell lung cancer: long-term results of a multi-institutional, phase II, randomized study. J Clin Oncol.

[51] Iyengar P, Wardak Z, Gerber DE (2018). Consolidative radiotherapy for limited metastatic non-small-cell lung cancer: a phase 2 randomized clinical trial. JAMA Oncol.

[52] Palma DA, Olson R, Harrow S (2020). Stereotactic ablative radiotherapy for the comprehensive treatment of oligometastatic cancers: long-term results of the SABR-COMET phase II randomized trial. J Clin Oncol.

[53] Harrow S, Palma DA, Olson R (2022). Stereotactic Radiation for the Comprehensive Treatment of Oligometastases (SABR-COMET)—extended long-term outcomes. Int J Radiat Oncol Biol Phys.

[54] Ost P, Reynders D, Decaestecker K (2018). Surveillance or metastasis-directed therapy for oligometastatic prostate cancer recurrence: a prospective, randomized, multicenter phase II trial. J Clin Oncol.

[55] Loi M, Alifano M, Scorsetti M (2021). Judging a fish by its ability to climb a tree? A call for novel endpoints in the appraisal of ablative local treatments of oligometastatic cancer. Oncologist.

[56] Mitchell KG, Farooqi A, Ludmir EB (2021). Pulmonary resection is associated with long-term survival and should remain a therapeutic option in oligometastatic lung cancer. J Thorac Cardiovasc Surg.

[57] Qiu H (2019). Stereotactic body radiation therapy versus metastasectomy for oligometastases. J Thorac Dis.

[58] Londero F, Grossi W, Morelli A (2020). Surgery versus stereotactic radiotherapy for treatment of pulmonary metastases. A systematic review of literature. Future Sci OA.

